# PlantMirP-Rice: An Efficient Program for Rice Pre-miRNA Prediction

**DOI:** 10.3390/genes11060662

**Published:** 2020-06-18

**Authors:** Huiyu Zhang, Hua Wang, Yuangen Yao, Ming Yi

**Affiliations:** 1 Department of Physics, College of Science, Huazhong Agricultural University, Wuhan 430070, China; zhanghuiyu@webmail.hzau.edu.cn; 2 School of Basic Medical Science, Hubei University of science and technology, Xianning 437100, China; whlei@alumni.hust.edu.cn; 3 School of Mathematics and Physics, China University of Geosciences, Wuhan 430074, China; mingyi@cug.edu.cn

**Keywords:** rice, microRNA, prediction, random forest, knowledge-based energy feature

## Abstract

Rice microRNAs (miRNAs) are important post-transcriptional regulation factors and play vital roles in many biological processes, such as growth, development, and stress resistance. Identification of these molecules is the basis of dissecting their regulatory functions. Various machine learning techniques have been developed to identify precursor miRNAs (pre-miRNAs). However, no tool is implemented specifically for rice pre-miRNAs. This study aims at improving prediction performance of rice pre-miRNAs by constructing novel features with high discriminatory power and developing a training model with species-specific data. PlantMirP-rice, a stand-alone random forest-based miRNA prediction tool, achieves a promising accuracy of 93.48% based on independent (unseen) rice data. Comparisons with other competitive pre-miRNA prediction methods demonstrate that plantMirP-rice performs better than existing tools for rice and other plant pre-miRNA classification.

## 1. Introduction

MicroRNAs (miRNAs) are an important type of short (approximately 20–24 nucleotides (nt)) small non-coding RNA (sRNA), and they are involved extensively in post-transcriptional regulation of gene expression in animals, plants, and viruses [1]. In plants, the primary transcript of miRNA gene (pri-miRNA) is mainly transcribed from intergenic regions of the genome by RNA polymerase II, and then pri-miRNA is cleaved into miRNA precursor (pre-miRNA) with characteristic stem–loop (hairpin) structure. Subsequently, pre-miRNA, which is exported to the cytoplasm under the action of HASTY protein, is cleaved by Dicer-like (DCL) enzyme into a miRNA duplex, consisting of a miRNA and miRNA* strand. miRNA duplex is further processed into mature miRNA in the cytoplasm. Finally, mature miRNA is included into RNA-induced silencing complex (RISC), and it then mediates the degradation or transcription inhibition of messenger RNA (mRNA) through the principle of complementary base pairing [2,3,4,5].

It has been confirmed that plant miRNAs are crucial regulators in plant growth, development, and stress resistance [6,7]. Particularly, *Oryza sativa* L. is an important crop and staple food for Asian countries, thus *Oryza sativa* miRNAs attract much attention. As a typical example, Wang et al. validated that miR164a, as a general negative regulator, is involved in rice immunity against the blast fungus by targeting OsNAC60. Furthermore, they argued that the miR164a/OsNAC60 module may be considered as a common immune regulator for diverse pathogens [8]. Moreover, yield of rice can be greatly increased by shaping inflorescence architecture through blocking miR396b with direct induction of the OsGRF6 gene (miR396b–OsGRF6 module) [9]. The effects of miR396c–OsGRF4-OsGIF1 regulatory module on grain size and yield of rice can be confirmed [10]. In addition, Swetha et al. revealed that major domestication-related phenotypes are related to loss of miRNA-mediated laccase silencing in Indian rice [11]. Transgenic microRNA-14 rice has been shown to have high resistance to rice stem borer [12].

Identification of miRNAs is a foundation for dissecting their regulatory functions. Traditionally, identifying miRNAs with experimental methods is inevitably time-consuming, cost-expensive, and even leads to many miRNAs being missed [13]. Next-generation sequencing technology has made it possible to identify miRNAs in genome-scale with high sensitivity. Currently, miRNAs are identified based on deep-sequencing technology followed by bioinformatics analyses and/or wet methods, such as Northern blot analyses and qPCR assay. Identification of miRNAs from deep-sequencing reads is almost exclusively based on identification of characteristic hairpin structures of pre-miRNA sequences. The pre-miRNA sequences are obtained by firstly mapping short sequences to genome, and then searching those loci that may produce stable stem–loop structures [14,15,16,17,18]. However, in order to improve true positive rate, many mapping loci with low density of read coverage may be directly discarded even if their flanking sequences can perfectly form hairpin structures. Therefore, these miRNA biogenesis-based approaches may miss some low abundance miRNAs [14,15,16,17,18].

Compared with the former methods, machine-learning-based methods do not require genomic information and expression information, and mainly leverage sequence and structure features of pre-miRNAs. Thus, machine-learning-based methods can be used for de novo prediction of miRNAs, i.e., without using a reference genome. In fact, some of these tools have been successfully used to distinguish pre-miRNAs from other RNA sequences [19,20,21,22,23]. Although, like animal pre-miRNAs, plant pre-miRNAs also have the stem–loop structures, secondary structure of plant pre-miRNAs is more complicated than that of animal pre-miRNAs, which makes plant pre-miRNA prediction more difficult. This may be a reason why few of prediction tools are designed specifically for plant pre-miRNAs. In 2010, Xuan et al. constructed a support vector machine-based (SVM-based) classifier (PlantMiRNAPred) with positive pre-miRNAs from eight plant species. The PlantMiRNAPred SVM performs excellently in the classification of real and pseudo plant pre-miRNAs [24]. In addition, other tools have also be developed for plant pre-miRNA detection, such as random forest-based HuntMi [25], decision tree-based miRNAprediction [26], and SVM-based miPlantPreMat [5]. Previously, we also developed plantMirP for prediction of plant pre-miRNAs by incorporating five knowledge-based energy features with 48 sequence and structure features.

Although some efforts have contributed to this area, no tool has been implemented specifically for rice pre-miRNA prediction. We argue that the performance of plant pre-miRNA prediction can be further improved if species-specific information embedded in sequences are artfully extracted and characterized by well-constructed features. To do this, we present a new set of knowledge-based energy features, then merge them with other features carefully selected from published studies to form a feature set. Based on rice pre-miRNAs from the miRBase database, we designed a random forest-based classifier of plantMirP-rice (hereinafter called riceMirP) to specifically predict rice pre-miRNAs. The riceMirP exhibits a very promising performance: an accuracy of 93.48%, sensitivity of 87.91%, specificity of 98.15%, and Mathew’s correlation coefficient of 0.8710 based on independent (unseen) rice data. 

## 2. Materials and Methods 

### 2.1. Data Preparation

After removing sequences containing non-AUCG nucleotides, 604 *Oryza sativa* pre-miRNAs were obtained from the miRBase database (release 22) (http://www.mirbase.org/) [27] and considered as a positive dataset. We randomly selected 422 real pre-miRNAs as positive training samples and used the others as independent positive testing samples. According to the existing method, negative datasets were produced from *Oryza sativa* protein coding sequences (CDSs) downloaded from the PlantGDB database (http://www.plantgdb.org/). To be specific, all CDS sequences of *Oryza sativa* were joined together to form a non-overlapping long sequence. Non-overlapping segments were extracted by fragmenting this long CDS sequence. The secondary structures of extracted segments and real rice pre-miRNAs were predicted by using RNAfold software (http://rna.tbi.univie.ac.at/cgi-bin/RNAWebSuite/RNAfold.cgi) with the default parameters. The extracted segments are referred to as pseudo pre-miRNAs if they have hairpin structures similar to that of the pre-miRNAs. Furthermore, in order to ensure high similarity between pseudo and real pre-miRNAs, two criteria for randomly selecting pseudo pre-miRNAs are that: (1) the number of paired bases (including GU wobble pairs) in the secondary structures of pseudo pre-miRNAs should not be lower than the minimum number of paired bases in the secondary structures of real pre-miRNAs and (2) the folding free energy of pseudo pre-miRNAs should not be higher than the maximum of the folding free energy of the real pre-miRNAs. Finally, 502 pseudo pre-miRNAs were randomly selected as negative training samples, and 216 pseudo pre-miRNAs as independent negative testing samples.

### 2.2. Feature Extraction

**Figure 1:**
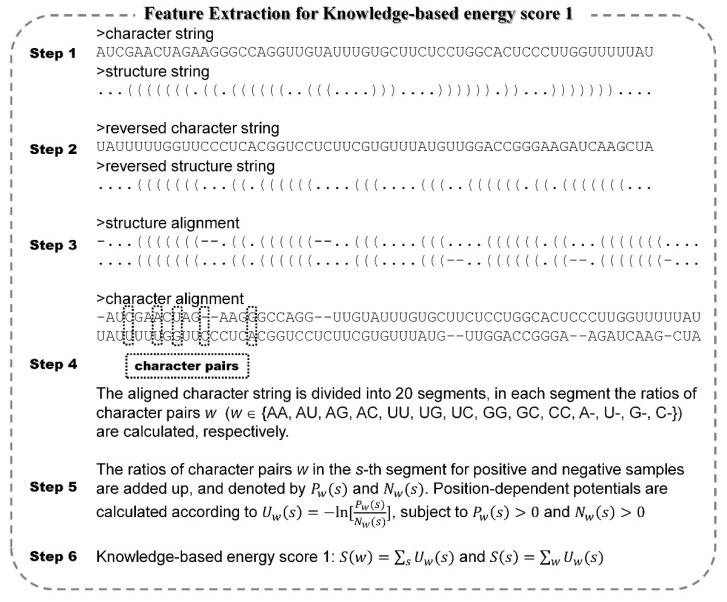
The flowchart of feature extraction for knowledge-based energy score 1.

The sequences (character strings) of real and pseudo pre-miRNAs are submitted into RNAfold to predict secondary structures (structure strings) with the default parameters (Figure 1). The character strings and structure strings of real and pseudo pre-miRNAs are reversed to obtain the corresponding reversed strings (Figure 1). As previously mentioned, in the predicted secondary structure, paired or unpaired nucleotides indicated by “(“ in 5′ end and “)” in 3′ end are indistinguishably represented by “(“ [20,28,29]. Then, the Needleman–Wunsch algorithm is used to align structure strings and the corresponding reversed structure strings. The symbol of “-” is used to fill the gap between the aligned structure strings (Figure 1). The aligned character strings are obtained according to the positions of “-” in the aligned structure strings (Figure 1). The aligned character strings are divided into 20 segments and the ratios of character pair *w* (*w* ∈ {AA, AU, AG, AC, UU, UG, UC, GG, GC, CC, A-, U-, G-, C-}) are calculated in each segment. The ratios of character pair *w* in the *s*-th segment for positive and negative samples are added up, respectively, and denoted by $P_{w}\left(s\right)$ and $N_{w}\left(s\right)$ Finally, position-dependent potentials $U_{w}\left(s\right)$ of character pair *w* in the s-th segment are calculated according to formula below:

(1) $$U_{w}\left(s\right)=-\ln\left[\frac{P_{w}\left(s\right)}{N_{w}\left(s\right)}\right]$$

Here $P_{w}\left(s\right)$ and $N_{w}\left(s\right)$ are required to be larger than 0. *w* runs through all possible character pairs, and *s* runs through all segments. Finally, a set of energy scores for a given sample is obtained according to the following formulae:

(2) $$S\left(w\right)=\sum_{s}U_{w}\left(s\right)$$

(3) $$S\left(s\right)=\sum_{w}U_{w}\left(s\right)$$

These energy scores of *S*(*w*) and *S*(*s*) are known as the knowledge-based energy score 1 (hereinafter called energy score 1) in order to distinguish them from the pre-existing knowledge-based energy features presented in our previous study [29]. The flowchart of feature extraction for knowledge-based energy score 1 is displayed in Figure 1.

### 2.3. Performance Evaluation

In a classification problem, four measures of the sensitivity (Se), specificity (Sp), accuracy (Ac), and Mathew’s correlation coefficient (MCC), are widely used to evaluate the prediction model, and they are calculated according to the following definitions [30,31,32]:

(4) $$\mathrm{Se}=\frac{TP}{TP+FN}$$

(5) $$\mathrm{Sp}=\frac{TN}{TN+FP}$$

(6) $$\mathrm{Ac}=\frac{TP+TN}{TP+FP+TN+FN}$$

(7) $$\mathrm{MCC}=\frac{\left(TP\;\;TN\right)-\left(FN\;\;FP\right)}{\sqrt{\left(TP+FN\right)\;\;\left(TN+FP\right)\;\;\left(TP+FP\right)\;\;\left(TN+FN\right)}}$$

Here TP (true positive) and FP (false positive) are the number of correctly- and incorrectly-predicted samples in positive samples, while TN (true negative) and FN (false negative) are the number of correctly- and incorrectly-predicted samples in negative samples. The receiver operating characteristic (ROC) curve is plotted for visualizing classification performance. Then, the area under the receiver operating characteristic (AUC) curve is used as a comprehensive measure to evaluate the classification algorithm.

### 2.4. Input, Output, Dependencies, Platforms, and Application Scenarios

RiceMirP, a random forest-based classifier, is implemented in Perl (v5.24.1) and R (v3.2.2), with the recommended versions in parentheses. The Random Forest algorithm is implemented by the *randomForest* R package (v4.6–14). RiceMirP is available as a stand-alone package where all necessary scripts and data (including data used in this study) are contained. The local package of riceMirP is freely available to the academic community at https://github.com/yygen89/riceMirP. RiceMirP can run on both Windows and Linux platforms. RiceMirP is designed in an easy-to-use manner and is an installation-free software. Before running riceMirP, the dependencies of Perl, R, and *randomForest* R package are required to be preinstalled in local machines. RiceMirP requires three FASTA-formatted input files: the first two files containing nucleotide sequences of positive and negative training samples, respectively, and the last one containing nucleotide sequences of testing samples. The output file of riceMirP includes the three-column contents of the identifier, predicted label (positive or negative), and corresponding score for each testing sample. Please make sure that the identifier for each sequence in FASTA-formatted files is unique. Generally, a higher score indicates that the testing sample is more likely to be a predicted label. RiceMirP can be applied to a sequence classification problem—for a given sequence, what is the likelihood of this given sequence being positive. For miRNA prediction from small RNA sequencing data, riceMirP, in conjunction with another kind of miRNA biogenesis-based approach, can further reduce the false-positive rate. 

## 3. Results

### 3.1. The Algorithm for the Prediction of Rice Pre-miRNAs

As we all know, for machine-learning-based classification and prediction, it is a quite crucial and challenging task to extract appropriate features to train a model. In our previous study, a set of knowledge-based energy features was constructed by tactfully combining a widely-used *k*-mer scheme in bioinformatics with distance-dependent potential in statistical physics [29]. In addition, knowledge-based energy features have been demonstrated to have very high discriminatory power [29], which suggests that relative position (or distance distribution) information of *k*-mer pairs is very valuable. In this study, we extend the distance-dependent potential presented previously [29] to position-dependent potential, and then construct a new set of knowledge-based energy features (energy score 1) (see details in Materials and Methods). In addition to the 34 novel features presented here, in order to further improve prediction performance, we also collect 49 sequence and structure features from previously-published studies. Full features used in riceMirP are listed in Table 1.

**Figure 2:**
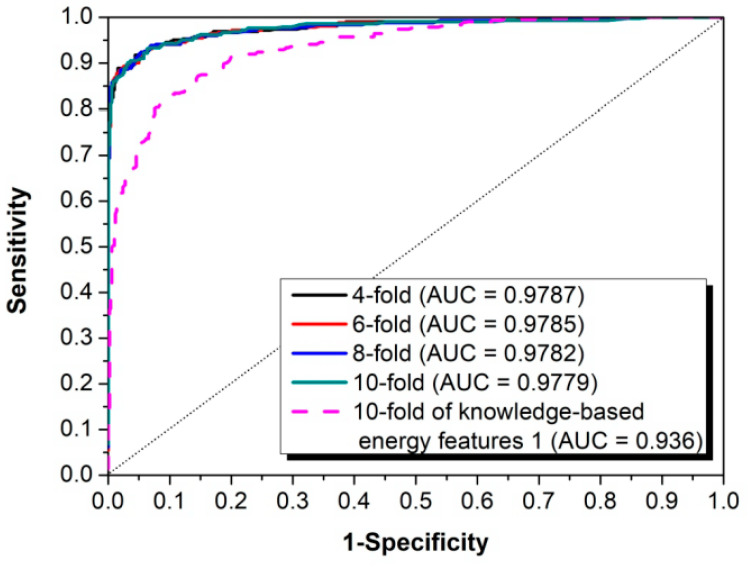
Receiver operating characteristic (ROC) curves from riceMirP prediction performance based on training dataset of riceMirP.

**Figure 3:**
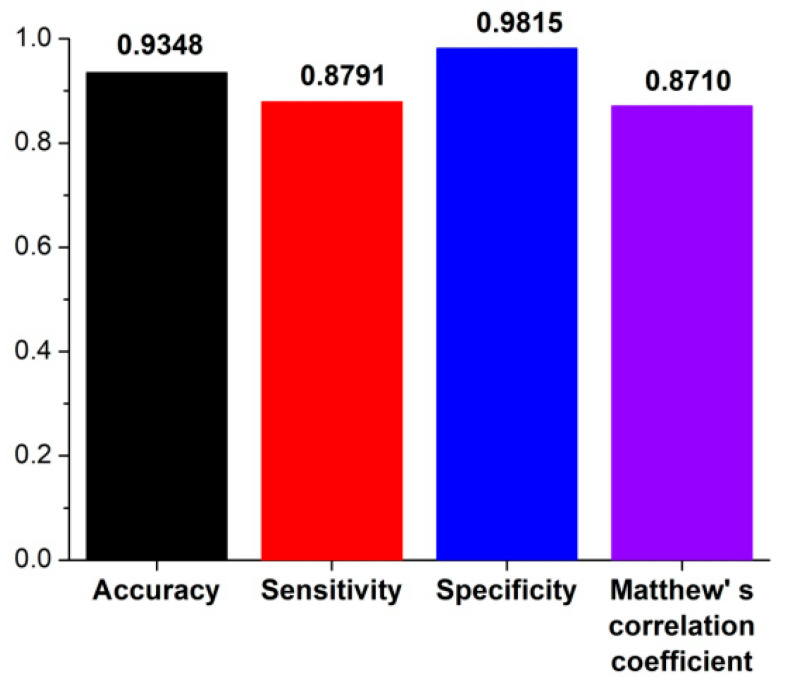
Sensitivity (Se), specificity (Sp), accuracy (Ac), and Matthew’s correlation coefficient (MCC) of riceMirP based on independent (unseen) testing dataset.

In order to train the prediction model, 422 known *Oryza sativa* pre-miRNAs (the positive training dataset) and 502 “pseudo” pre-miRNAs (the negative training dataset) were merged together to obtain a training dataset (see details in Materials and Methods). Based on this training dataset, 4-, 6-, 8-, and 10-fold cross-validations (CVs) were performed to evaluate performance of riceMirP. The ROC curves of 4-, 6-, 8-, and 10-fold CVs overlapped almost completely (Figure 2), which shows that riceMirP is very robust. Furthermore, the AUC values of 4-, 6-, 8-, and 10-fold CVs were 0.9787, 0.9785, 0.9782, and 0.9779, respectively (Figure 2), which indicates that riceMirP is a very promising tool. It is worth noting that if only using knowledge-based energy score 1 (including 34 features), the AUC value of the 10-fold CV was still as high as 0.936 (Figure 2). This result demonstrates that the above-mentioned features of energy score 1 constructed based on position-dependent potential have very high discriminating power. Moreover, an independent (unseen) testing dataset including 182 positive and 216 negative samples was randomly selected to evaluate the prediction performance of riceMirP (see details in Materials and Methods). As shown in Figure 3, riceMirP with full features had a promising Ac of 0.9348, Se of 0.8791, Sp of 0.9816, and MCC of 0.8710.

**Figure 4:**
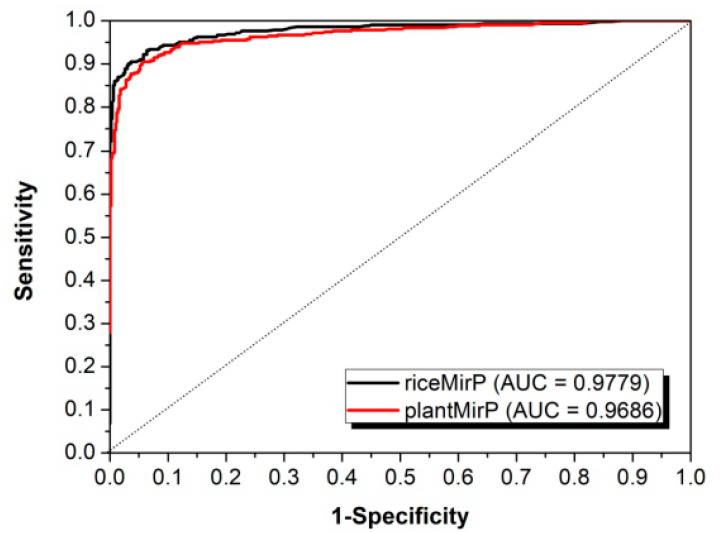
Comparison of riceMirP with plantMirP based on training dataset of riceMirP.

We compared riceMirP to the state-of-the-art plantMirP, which was recently designed especially for prediction of plant pre-miRNAs. Based on the same training dataset of riceMirP, the 10-fold CV was implemented for riceMirP and plantMirP, respectively. Figure 4 shows that the AUC values of riceMirPp and plantMirP were 0.9779 and 0.9686. Therefore, riceMirP performs better to plantMirP in rice pre-miRNA classification.

### 3.2. Comparison with Other Competitive Methods

**Figure 5:**
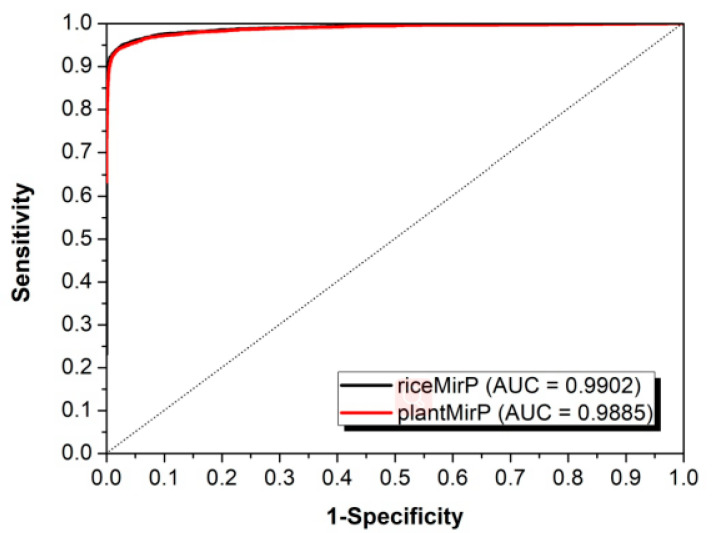
Comparison of riceMirP with plantMirP based on the training dataset of plantMirP.

In order to test whether riceMirP can be used for other plant pre-miRNA prediction, we compared riceMirP with competitive pre-miRNA prediction methods. In addition, to avoid any possible bias from data used in riceMirP, all comparisons were performed based on the data of other tools or the data from the third party. We firstly compared riceMirP to plantMirP based on the training dataset of plantMirP. Likewise, the performance comparison between two tools was visualized by the ROC curve of 10-fold CV (Figure 5). It is evident that riceMirP is slightly superior to plantMirP in plant pre-miRNA prediction.

**Figure 6:**
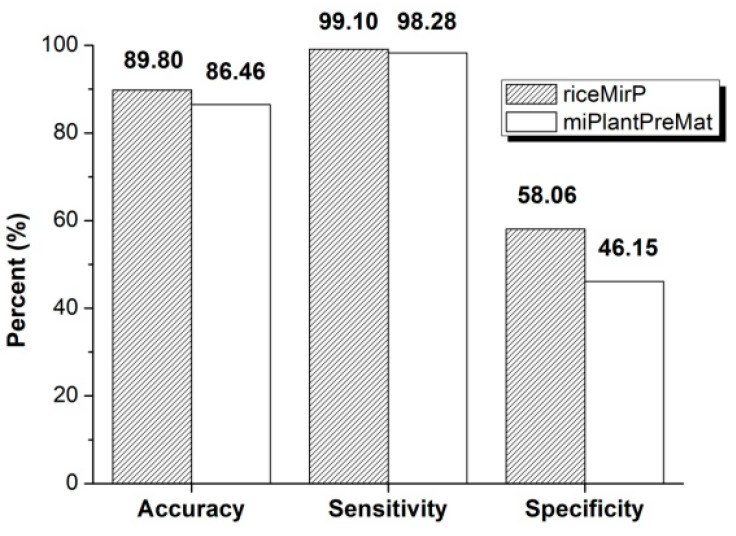
Accuracy (Ac), sensitivity (Se), and specificity (Sp) of riceMirP and miPlantPreMat based on the datasets from miPlantPreMat and PlantMiRNAPred.

Further, we compared riceMirP with miPlantPreMat, which is an SVM-based classifier for identifying plant pre-miRNAs and the corresponding mature miRNAs. Similarly, in order to avoid potential effects from dataset, we firstly used the dataset (“mirPlantPre19_single.txt” & “negData.txt”) of miPlantPreMat to train a prediction model for riceMirP. Then, the dataset (“mirPlantPre20_single.txt”) of miPlantPreMat was considered as a positive testing dataset, and submitted into miPlantPreMat and riceMirP for prediction, respectively. Because there is no negative testing dataset in the package of miPlantPreMat, the negative samples from third-party (i.e., PlantMiRNAPred) were collected as an independent negative testing dataset, and then directly submitted into riceMirP and miPlantPreMat for prediction. In this way, the Ac, Sp, and Se values of riceMirP and miPlantPreMat were obtained, respectively. Obviously, riceMirP achieved better classification performance than miPlantPreMat in plant pre-miRNA prediction (Figure 6).

**Figure 7:**
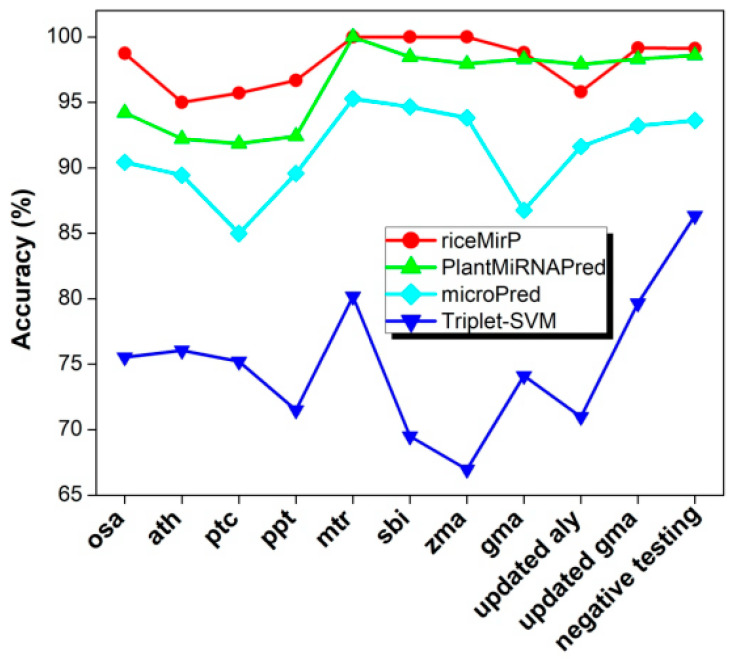
Comparison of riceMirP with triplet-support vector machine (SVM), microPred, and PlantMiRNAPred based on the training and testing datasets from PlantMiRNAPred. The classification results reported previously are directly used for comparison [24].

Finally, we compared riceMirP with triplet-SVM, microPred, and PlantMiRNAPred. In particular, PlantMiRNAPred is designed specifically for prediction of plant pre-miRNAs, and achieves >90% accuracy on multiple plant datasets. Likewise, the training dataset (“train_negative_980_seq.txt” & “train_positive_980_seq.txt”) of PlantMiRNAPred was used as a training dataset to train model for riceMirP. Then, 11 testing datasets of PlantMiRNAPred were submitted into riceMirP to calculate prediction accuracy. These testing datasets of PlantMiRNAPred included three parts: the known plant pre-miRNAs from eight species, which were used for evaluating the ability of identifying the real pre-miRNAs; the 1142 negative testing samples, which were used for testing the ability of identifying the pseudo hairpins; and the updated dataset, which were used to observe the ability of discovering new plant pre-miRNAs. Because there is no stand-alone version of PlantMiRNAPred, and the web-server of PlantMiRNAPred was not available, classification results reported previously [24] are directly adopted for comparison. For all testing datasets (except the “updated aly”), the accuracies of riceMirP were higher than those of PlantMiRNAPred (Figure 7). Furthermore, the overall accuracy of riceMirP was much better than those of microPred and triplet-SVM (Figure 7). The above-mentioned results indicate that the schemes of feature extraction and algorithm presented here are universal and are not limited to rice.

## 4. Conclusions

In this study, a promising random forest-based classifier, riceMirP, was constructed specifically for predicting rice pre-miRNAs by combining 34 novel knowledge-based energy features with 49 other existing sequence and structure features extracted from published studies. Particularly, riceMirP was superior to the state-of-the-art plantMirP in rice pre-miRNA prediction, which suggests that it is useful to construct a prediction tool specifically for rice pre-miRNAs. In addition, the extensive comparisons with existing pre-miRNA prediction methods, such as plantMirP, miPlantPreMat, PlantMiRNAPred, triplet-SVM, and microPred demonstrated that riceMirP also exhibits higher classification performance in other plant pre-miRNA prediction. Moreover, these above-mentioned results also illustrate that the novel knowledge-based energy features (i.e., energy score 1) proposed here have very high discriminatory power, and that the scheme of feature extraction presented here is universal and is not limited to rice. Taken together, the results obtained in this study might be beneficial for subsequent researches. 

## Figures and Tables

**Table 1:** Full features used in plantMirP-rice (riceMirP)

No.	Feature	Description	Origin
1–34	Energy score 1	Obtained from position-dependent potentials with character pair *w.*	Novel
35	Energy score 2	Obtained from distance-dependent potentials with 3-mer pairs.	plantMirP
36–45	Ratio of Unpaired bases in sub-region	The secondary structure is divided into 10 parts, and the ratio in each part is calculated.	plantMirP
46	Size of biggest bulge	A bulge contains at least three adjacent unpaired bases.	plantMirP
47	n_loops/L	n_loops denotes the number of loops, L is the length of sequence.	plantMirP
48	n_stems/L	A stem consists of at least three continuous paired bases.	plantMirP
49	%(|G| + |C|)	(|G| + |C|)/L * 100, here |X| denotes the number of X in sequence.	miPred
50–65	%XY	|XY|/(L − 1) * 100, |XY| is number of dinucleotide XY in sequence.	miPred
66	dG	MFE/L, MFE is minimum of free energy of the secondary structure.	miPred
67	MFE1	(MFE/L)/%(|G| + |C|)	miPred
68	MFE2	(MFE/L)/n_stems	miPred
69	dP = tot_bases/L	tot_bases is number of base pairs in the secondary structure.	miPred
70	MFE3	(MFE/L)/n_loops	microPred
71–73	|X − Y|/L	|X − Y| is the number of base pairs, (X − Y)∈[(A − U), (G − C), (G − U)]	microPred
74	Avg_bp_stem	tot_bases/n_stems, n_stems denotes the number of stems.	microPred
75–77	%(X − Y)/n_stems	%(X − Y) = |X − Y|/tot_bases	microPred
78	pb/nb	The ratio of paired nucleotides to unpaired nucleotides.	miRD
79	MCPN	Maximum of consecutive paired nucleotides.	ZmirP [33]
80	n_bulges/L	n_bulges is the total number of bulges in the secondary structure.	ZmirP
81	Avg_bp_stem	The ratio of number of base pairs to n_stems.	ZmirP
82	MFE4	dG/tot_bases	ZmirP
83	MFE5	dG/n_bulges	ZmirP

## References

[1] He L., Hannon G.J. (2004). MicroRNAs: Small RNAs with a big role in gene regulation. Nat. Rev. Genet..

[2] Teune J.-H., Steger G. (2010). NOVOMIR: De Novo Prediction of MicroRNA-Coding Regions in a Single Plant-Genome. J. Nucleic Acids.

[3] Ding Y., Tao Y., Zhu C. (2013). Emerging roles of microRNAs in the mediation of drought stress response in plants. J. Exp. Bot..

[4] Voinnet O. (2009). Origin, Biogenesis, and Activity of Plant MicroRNAs. Cell.

[5] Meng J., Liu D., Sun C., Luan Y.-S. (2014). Prediction of plant pre-microRNAs and their microRNAs in genome-scale sequences using structure-sequence features and support vector machine. BMC Bioinform..

[6] Mallory A.C., Vaucheret H. (2006). Functions of microRNAs and related small RNAs in plants. Nat. Genet..

[7] Navarro L., Dunoyer P., Jay F., Arnold B., Dharmasiri N., Estelle M., Voinnet O., Jones J.D.G. (2006). A Plant miRNA Contributes to Antibacterial Resistance by Repressing Auxin Signaling. Science.

[8] Wang Z., Xia Y., Lin S., Wang Y., Guo B., Song X., Ding S., Zheng L., Feng R., Chen S. (2018). Osa-miR164a targetsOsNAC60and negatively regulates rice immunity against the blast fungusMagnaporthe oryzae. Plant J..

[9] Gao F., Wang K., Liu Y., Chen Y., Chen P., Shi Z., Luo J., Jiang D., Fan F., Zhu Y. (2015). Blocking miR396 increases rice yield by shaping inflorescence architecture. Nat. Plants.

[10] Li S., Gao F., Xie K., Zeng X., Cao Y., Zeng J., He Z., Ren Y., Li W., Deng Q. (2016). The OsmiR396c-OsGRF4-OsGIF1 regulatory module determines grain size and yield in rice. Plant Biotechnol. J..

[11] Swetha C., Basu D., Pachamuthu K., Tirumalai V., Nair A., Prasad M., Shivaprasad P.V. (2018). Major Domestication-Related Phenotypes in Indica Rice Are Due to Loss of miRNA-Mediated Laccase Silencing. Plant Cell.

[12] He K., Xiao H., Sun Y., Ding S., Situ G., Li F. (2018). Transgenic microRNA-14 rice shows high resistance to rice stem borer. Plant Biotechnol. J..

[13] Berezikov E., Cuppen E., Plasterk R.H.A. (2006). Approaches to microRNA discovery. Nat. Genet..

[14] Xie F., Xiao P., Chen N., Xu L., Zhang B. (2012). miRDeepFinder: A miRNA analysis tool for deep sequencing of plant small RNAs. Plant Mol. Boil..

[15] Friedländer M.R., Chen W., Adamidi C., Maaskola J., Einspanier R., Knespel S., Rajewsky N. (2008). Discovering microRNAs from deep sequencing data using miRDeep. Nat. Biotechnol..

[16] Friedländer M.R., Mackowiak S., Li N., Chen W., Rajewsky N. (2011). miRDeep2 accurately identifies known and hundreds of novel microRNA genes in seven animal clades. Nucleic Acids Res..

[17] Yang X., Li L. (2011). miRDeep-P: A computational tool for analyzing the microRNA transcriptome in plants. Bioinformatics.

[18] An J., Lai J., Lehman M.L., Nelson C.C. (2012). miRDeep: An integrated application tool for miRNA identification from RNA sequencing data. Nucleic Acids Res..

[19] Morgado L., Johannes F. (2019). Computational tools for plant small RNA detection and categorization. Brief. Bioinform..

[20] Xue C., Li F., He T., Liu G.-P., Li Y., Zhang X. (2005). Classification of real and pseudo microRNA precursors using local structure-sequence features and support vector machine. BMC Bioinform..

[21] Batuwita R., Palade V. (2009). microPred: Effective classification of pre-miRNAs for human miRNA gene prediction. Bioinformatics.

[22] Yousef M., Nebozhyn M., Shatkay H., Kanterakis S., Showe L.C., Showe M.K. (2006). Combining multi-species genomic data for microRNA identification using a Naive Bayes classifier. Bioinformatics.

[23] Chang D.T.-H., Wang C.-C., Chen J.-W. (2008). Using a kernel density estimation based classifier to predict species-specific microRNA precursors. BMC Bioinform..

[24] Xuan P., Guo M., Liu X., Huang Y., Li W. (2011). PlantMiRNAPred: Efficient classification of real and pseudo plant pre-miRNAs. Bioinformatics.

[25] Gudyś A., Szcześniak M., Sikora M., Makałowska I. (2013). HuntMi: An efficient and taxon-specific approach in pre-miRNA identification. BMC Bioinform..

[26] Williams P.H., Eyles R., Weiller G. (2012). Plant MicroRNA Prediction by Supervised Machine Learning Using C5.0 Decision Trees. J. Nucleic Acids.

[27] Kozomara A., Griffiths-Jones S. (2013). miRBase: Annotating high confidence microRNAs using deep sequencing data. Nucleic Acids Res..

[28] Zhang Y., Yang Y., Zhang H., Jiang X., Xu B., Xue Y., Cao Y., Zhai Q., Zhai Y., Xu M. (2011). Prediction of novel pre-microRNAs with high accuracy through boosting and SVM. Bioinformatics.

[29] Yao Y., Ma C., Deng H., Liu Q., Zhang J., Yi M. (2016). PlantMirP: An efficient computational program for the prediction of plant pre-miRNA by incorporating knowledge-based energy features. Mol. BioSyst..

[30] Zhao Q., Yang Y., Ren G., Ge E., Fan C. (2019). Integrating Bipartite Network Projection and KATZ Measure to Identify Novel CircRNA-Disease Associations. IEEE Trans. NanoBiosci..

[31] Liu H., Ren G., Chen H., Liu Q., Yang Y., Zhao Q. (2020). Predicting lncRNA–miRNA interactions based on logistic matrix factorization with neighborhood regularized. Knowl. Based Syst..

[32] Liu Z., Ren J., Cao J., He J., Yao X., Jin C., Xue Y. (2012). Systematic analysis of the Plk-mediated phosphoregulation in eukaryotes. Brief. Bioinform..

[33] Yao Y., Ma L., Jia Q., Deng W., Liu Z., Zhang Y., Ren J., Xue Y., Jia H., Yang Q. (2014). Systematic characterization of small RNAome during zebrafish early developmental stages. BMC Genom..

